# Supplementary material for: Annotation of snoRNA abundance across human tissues reveals complex snoRNA-host gene relationships
Source: Genome Biol. 2021 Jun 4;22:172. doi: 10.1186/s13059-021-02391-2 (PMC8176728; doi:10.1186/s13059-021-02391-2)
Supplement: Supplementary file 3 — Additional file 3. Peer review history. [file 13059_2021_2391_MOESM3_ESM.docx]

**1^st^ round**

**Reviewer 1**

The manuscript by Fafard-Couture et al. describes an RNA-seq study of RNA isolated from seven human tissues with emphasis on the expression levels of intron-encoded guide RNAs and their host genes. A main point is the definition of two abundance classes of snoRNAs - uniformly expressed (UE) and tissue enriched (TE) - defined by a coefficient of variation (CV) calculated based on abundance across all studied tissues and separated by a threshold value. The two abundance classes are described as having differences in genomic context, conservation, expression pattern, and function (to the extent that function is known). This leads to a model describing four different genomic settings for host gene and snoRNA expression.

This is mainly a confirmatory study providing supportive evidence to existing literature and extending observations made e.g. in mouse or cell culture models to human tissues. I am a little disappointed about the insufficient referencing and the inflated language ("great need", p. 2, l. 24, "blueprint for snoRNA function", p. 2, l. 25, "reliable portrait" p. 18, l. 385, "serve as a valuable source" p. 18, l. 390), but find overall that the study is a valuable contribution.

Major concerns

1. The authors present their data as having "little or no structural bias". I strongly object to this although I agree that the use TGIRT-Seq and improved sequence assignment will help overcome some sources of bias. The experimental pipeline involves many steps that will introduce a bias, including purification and enzymatic steps. To give a few examples, using the RNeasy Mini kit introduces a bias compared to the classical purification of a small RNA size fraction by gradient centrifugation. Here, it is followed by ribosomal RNA depletion using "Ribo-Zero Gold" leading to an "rRNA-free RNA preparation" (p. 23, l. 511). The authors should document this step. Was the rRNA fraction analyzed for co-depletion of snoRNAs? It is well-known that some snoRNAs will stick to rRNA during purification due to extensive base pairing. In some cases, the oligos used for rRNA depletion will also bind to rRNA-targeting snoRNAs. Some snoRNAs are more susceptible to RNA fragmentation than others and several of the cloning steps are also known to introduce bias.

Besides the inherent problem in claiming bias-free sequencing data, the present study has no independent validation experiments, e.g. northerns of selected snoRNAs. Furthermore, when inspecting the expression levels in Table S4, there is considerable variation between replicates, including variation in relative snoRNA expression levels. Yet, the manuscript has no discussion of possible sources of variation. It should be mentioned and exemplified that the tissues comprise many cell types that probably have very different RNA profiles. Thus, biopsies may differ in cell composition and be a source of variation. If the authors insist that there is no experimental bias, is the observed variation within a range that is expected based on individual variation in expression levels? Inspection of the data in Table S4 leads to some puzzling observations. As an example, SNORD77 is one of many SNORDs expressed from Gas5. The levels in Table S4 are very low in all tissues and much lower than other Gas5 SNORDs. Yet, this SNORD guides methylation of position 1521 in the large subunit rRNA and literature shows that this position is fully methylated in both cultured cells and tissues, including brain and skeletal muscle. How is this explained?

2. The host gene encoded mRNAs have very different half-lives. For example, many host genes encode ribosomal protein mRNAs with much longer half-lives than most members of the heterogeneous group of other host genes. The same is probably also true for lncRNA-encoding host genes, although this is more difficult to consider due to complexity in splicing patterns and isoforms. The authors should take transcript half-lives into consideration in their discussions of correlations between guide RNA and host gene encoded mRNA and lncRNA expression levels.

The authors find support for uncoupling of snoRNA and host gene expression in studies of expression levels of AML and cell lines. However, it is typical of these models that the methylation system is unable to keep up with increased ribosome biogenesis, so these observations may have a more trivial explanation.

Other comments

Title and other places: The title "Dynamics and modulation of the human snoRNome" and sentences like "to follow the expression of both snoRNAs and HGs in seven healthy human tissues" (p. 5, l. 109) and "dynamics of brain and reproductive tissue snoRNomes ...." (p. 11, l. 244) suggest a time-variable in the study. I have been unable to find justification for this.

The use of "snoRNA" as a term throughout leads to the unfortunate situation that RNAs that are not nucleolar are labelled as such. The authors should be explicit about this or, preferably, use better terms, including the established gene names (e.g. SNORD and SNORA).

The authors should make clear early in the manuscript that they use of the term "expression" in the narrow sense, simply to mean "RNA levels" or, better, use more precise language.

p. 3., l. 49 (and other places): "Small nucleolar RNAs (snoRNAs) are a conserved family of mid-size non-coding RNA best characterized as regulators of ribosome biogenesis." There is no evidence that the majority of snoRNAs are "regulatory" or that the installation of a 2'OMe "leads to ribosome assembly" (l. 50). In fact, the literature suggests that most are dispensable. Along the same line, the de-regulation of snoRNA expression in cancer is mostly hypomethylation and can equally well be explained by an inability of the methylation mechanism to keep up with increased ribosome biogenesis. The authors should define their use of the term "regulatory" or, better, avoid using this term loosely.

p. 3, l. 54-60: Include acetylation guide RNAs for completion.

p. 4, l. 67: The authors should mention that the example of cis base pairing (ref. 11) may be a special case involved in a regulatory mechanism of snoRNA biogenesis.

p. 5, l. 98: The authors should provide evidence for claiming "Indeed, the highly stable and chemically modified structure of snoRNAs impair their reverse transcription, ...". In my opinion, snoRNAs (in particular box C/D) have a limited structural component (e.g. their antisense elements are exposed) and are stabilized by their associated proteins. Furthermore, the evidence for chemical modifications is very limited. Certainly, the evidence from the Zavolan lab is insufficient to support such a conclusion.

p. 9, l. 201: "...enrichment of the different genes" should be "gene transcripts" or "transcripts".

p. 13., l. 274-76: The sentence "This suggests that TE snoRNAs have a greater degree of regulatory freedom than the UE group that needs to take in consideration the constraints of the HG expression and function." is unclear to me. What is the meaning of "regulatory freedom"? It seems to me that the authors imply that host genes and snoRNAs (in this case they are most likely not nucleolar and should not be named as such) co-evolve. If so, the authors should provide evidence for this.

p. 14, l. 305-07: "The increased difference between anticorrelated snoRNAs and their HG suggests a negative regulatory loop by which the expression of snoRNAs represses that of the HG." This is highly speculative and should not appear in a results section. Furthermore, what exactly do the authors imply by a "regulatory loop". There is no overall correlation between SNORD levels and modification levels which suggests that SNORD levels not are stringently regulated (or that many SNORDs have additional functions). In several multi-SNORD host genes, the SNORDs have very different abundancies. How is this to be understood in relation to a correlation between SNORDs and host gene transcript abundancies?

p. 15, l. 334-341: This part was hard to follow. I am quite skeptical about the assignment of functions to many lncRNA and suggest that further arguments are included.

p. 16, l. 343: The authors should provide the reader with a short explanation of the background for the data on susceptibility to NMD and should specify if they used the data from the original publication (as referenced) or from the substantial corrigendum that appeared two years later.

p. 16, l. 360-365: The presentation of the results from ref. 19 on promoter structure is oversimplified. The paper describes two types of initiation sites (YR and YC). Typically each type is used more than once in a promoter. Thus, a promoter is not "single initiation", but "single type initiation" (see e.g. ref. 19, fig. 1b). This also implies that the term "Monolithic initiation" is incorrect! The drawing of the DI promoter in Fig. 6 is incorrect. YR and YC initiation sites are typically closely spaced in the core promoter region (Ref. 19, fig. 1c). In Fig. 6, the YC initiation is drawn much downstream of the YR site and the figure implies that this gives rise to a PTC, which is highly unlikely. A more plausible mechanism for the PTC is alternative splicing which in principle could be linked to different initiation complexes at the two types of TSS, but this is speculative at present.

p. 19, l. 423: I assume that the authors mean "positively correlated" and "single type transcription initiation site".

**Reviewer 2**

The article offers highly detailed classification of snoRNAs in a number of human tissues. Overall, the article is well-written and the categories into which the snoRNAs are divided are informative and well-supported. While it is undeniably a valuable resource, the article is purely descriptive. There is no experimental manipulation which would allow for determining the functions of the different snoRNAs.

1. There are certain speculative statements regarding the function of the snoRNAs this article is concerned with in the results section. While some of them are supported by present or previous data, others do not belong in a descriptive article such as this. It would be better to move these statements into the discussion. Examples:

Line 235 - 236: "Together, these data suggest that some snoRNAs may play specialized roles in the biology of brain and reproductive tissues."

Line 243 - 245: "These findings indicate that the distinct nature and dynamics of brain and reproductive tissue snoRNomes may reflect at least in part the clear difference in the proliferative state of the cells forming these two tissue types."

Line 266 - 269: "… indicating different functions associated with the two abundance classes." Etc.

2. The figures are crowded due to the limited spacing between the axes and the axes labels. Please adjust the figures so the axes labels are father removed from the axes themselves to improve readability.

3. More detail about the individuals the tissues originated from would be helpful. How many were male/female? What age?

4. What was the minimum RIN score for the samples?

5. Please report only the p-value in text, without the stars.

6. The percentages in Figure 1 A don't seem to correspond to the total numbers.

7. Please fix the x axis in Figure 2 C and Figure 4 B.

**Reviewer 3**

The authors report the application of an RNAseq variant to determine the abundances of snoRNAs across multiple human tissue samples. This leads to conclusions about the gene organization of different groups of snoRNA, which are classified on the basic of their relative accumulation in different tissues.

A considerable amount of data are presented, and the analyses appear to have been competently performed with appropriate statistical treatment. Overall, the results are quite descriptive but should be of value to the field. As a class snoRNAs have attracted considerable attention due to correlations with disease states, but their diversity has greatly complicated generalized conclusions. The work reported helps with this significant problem and I would be supportive of publication.

Minor points:

1. For DI vs SI and NMD sensitivity, it would be good to state how these were defined in the main text. These are features used a s major classifiers. Where gene lists simply taken from the references cited in Methods?

2. In several places there are sentences like: " snoRNAs are generally more expressed than their HG". Gene expression is often used to describe transcription rates. It would be clearer for the reader to distinguish transcription from accumulation, which is actually being measured. The latter is the product of transcription, processing and stability. Some discussion of this point would likely help the reader.

**Authors’ response**

Reviewer #1: The manuscript by Fafard-Couture et al. describes an RNA-seq study of RNA isolated from seven human tissues with emphasis on the expression levels of intron-encoded guide RNAs and their host genes. A main point is the definition of two abundance classes of snoRNAs - uniformly expressed (UE) and tissue enriched (TE) - defined by a coefficient of variation (CV) calculated based on abundance across all studied tissues and separated by a threshold value. The two abundance classes are described as having differences in genomic context, conservation, expression pattern, and function (to the extent that function is known). This leads to a model describing four different genomic settings for host gene and snoRNA expression.

This is mainly a confirmatory study providing supportive evidence to existing literature and extending observations made e.g. in mouse or cell culture models to human tissues. I am a little disappointed about the insufficient referencing and the inflated language ("great need", p. 2, l. 24, "blueprint for snoRNA function", p. 2, l. 25, "reliable portrait" p. 18, l. 385, "serve as a valuable source" p. 18, l. 390), but find overall that the study is a valuable contribution.

Response: We thank this reviewer for finding the study valuable. We believe that the insufficient referencing and inflated language noted by the reviewer stems from our focusing the scope of the paper on human tissues. Indeed, we believe that the language and referencing are appropriate when we consider the literature dealing with the sequencing of human tissues. However, to address this comment we have now included referencing to other species and adjusted the language accordingly.

Major concerns

1. The authors present their data as having "little or no structural bias". I strongly object to this although I agree that the use TGIRT-Seq and improved sequence assignment will help overcome some sources of bias. The experimental pipeline involves many steps that will introduce a bias, including purification and enzymatic steps. To give a few examples, using the RNeasy Mini kit introduces a bias compared to the classical purification of a small RNA size fraction by gradient centrifugation. Here, it is followed by ribosomal RNA depletion using "Ribo-Zero Gold" leading to an "rRNA-free RNA preparation" (p. 23, l. 511). The authors should document this step. Was the rRNA fraction analyzed for co-depletion of snoRNAs? It is well-known that some snoRNAs will stick to rRNA during purification due to extensive base pairing. In some cases, the oligos used for rRNA depletion will also bind to rRNA-targeting snoRNAs. Some snoRNAs are more susceptible to RNA fragmentation than others and several of the cloning steps are also known to introduce bias.

Response: We agree with the reviewer that although our approach using TGIRT-seq and improved read attribution at least partly alleviates some sources of bias and represents the current state of the art for high-throughput quantification of whole rRNAdepleted transcriptomes, the approach is not bias-free. We have reworded the sentence claiming ‘little or no structural bias’, indicating our approach as having ‘considerably less structural bias than standard approaches’ with appropriate references. The validity of this sequencing approach was already addressed by a number of previous publications by our group as well as other groups and is not the subject of this study that builds on a solid base of literature (Qin et al 2016 RNA; Nottingham et al 2016 RNA; Carrell et al 2018 NAR; Boivin et al RNA; Xu et al 2020 Sci Rep; Yao et al 2020 Elife). In addition, we would like to underline some of the evidence supporting the accuracy of our snoRNA quantification method:

A) The overall abundance hierarchy of snoRNA with respect to other RNA subtypes is maintained and reflects the data obtained using direct biochemical means as previously published (Boivin et al 2018 RNA).

B) The hierarchy of snoRNA as detected in the ribodepleted sequencing approach was also obtained using non-ribodepleted RT-qPCR and ddPCR showing that the ribodepletion has little impact on snoRNA rank (Boivin et al 2018 RNA, Deschamps- Francoeur et al 2019 Bioinformatics).

C) SnoRNAs targeting snRNAs, which would not be affected by rRNA depletion, are less abundant (on average abundant at 175 TPM across tissues) than snoRNAs targeting rRNAs (on average abundant at 540 TPM across tissues), underlining that rRNA-targeting snoRNAs are not underrepresented in our datasets.

D) As expected, snoRNAs targeting the most important regions of rRNA are amongst the most abundant snoRNAs in our datasets. For example, the snoRNAs targeting the peptidyl-transferase center are highly abundant across tissues with an average abundance of 662 TPM.

Finally, it should be noted that the absolute accuracy or any inherent bias of the sequencing method has no bearing on the main conclusion of this manuscript, which is based on the comparison between different tissues using the same sequencing method.

Besides the inherent problem in claiming bias-free sequencing data, the present study has no independent validation experiments, e.g. northerns of selected snoRNAs. Furthermore, when inspecting the expression levels in Table S4, there is considerable variation between replicates, including variation in relative snoRNA expression levels. Yet, the manuscript has no discussion of possible sources of variation. It should be mentioned and exemplified that the tissues comprise many cell types that probably have very different RNA profiles. Thus, biopsies may differ in cell composition and be a source of variation. If the authors insist that there is no experimental bias, is the observed variation within a range that is expected based on individual variation in expression levels? Inspection of the data in Table S4 leads to some puzzling observations. As an example, SNORD77 is one of many SNORDs expressed from Gas5. The levels in Table S4 are very low in all tissues and much lower than other Gas5 SNORDs. Yet, this SNORD guides methylation of position 1521 in the large subunit rRNA and literature shows that this position is fully methylated in both cultured cells and tissues, including brain and skeletal muscle. How is this explained?

Response: As indicated above, the accuracy and quality of our TGIRT based sequencing protocol was the principal subject of an earlier publication that clearly showed that abundance ranks obtained for snoRNAs by this sequencing method are reproduced by RT-qPCR and biochemical methods (Boivin et al 2018 RNA). Indeed, we have verified the overall distribution of RNA subtypes and inter-RNA species rank by comparison to earlier and published PCR. Accordingly, we have referred the readers to earlier studies validating the accuracy of this sequencing technique. In addition, it should be again noted that the important point here is not the absolute validity of the sequencing but the reproducibility of sequencing results across technical replicates, which was already established as highly reproducible (Boivin et al 2018 RNA). To address the issue of variation between replicates, we have now added sentences describing possible sources of variability between same tissue samples, at the beginning of the Results section. In addition, we have now referred the reader to Figure S1 that describes the capacity of our datasets to discriminate between tissues despite the inherent sources of variability.

The low expression level of SNORD77 (14 TPM on average across tissues) could be explained by the high abundance of SNORD80 (1235 TPM on average across tissues) which is predicted to target the same methylation position in the 28S rRNA.

2. The host gene encoded mRNAs have very different half-lives. For example, many host genes encode ribosomal protein mRNAs with much longer half-lives than most members of the heterogeneous group of other host genes. The same is probably also true for lncRNA-encoding host genes, although this is more difficult to consider due to complexity in splicing patterns and isoforms. The authors should take transcript halflives into consideration in their discussions of correlations between guide RNA and host gene encoded mRNA and lncRNA expression levels. The authors find support for uncoupling of snoRNA and host gene expression in studies of expression levels of AML and cell lines. However, it is typical of these models that the methylation system is unable to keep up with increased ribosome biogenesis, so these observations may have a more trivial explanation.

Response: The uncoupling of host and snoRNA expression may be achieved at different levels including post-transcriptionally by simple difference in RNA stability and difference in half-lives. However, systematic difference in the half-life of host and embedded snoRNA cannot explain why hosts with similar half-lives show varying degrees of coupling with their embedded snoRNA. In addition, inherent class or RNA subtype differences in half-lives of the host gene do not explain why snoRNAs embedded in introns of the same host have different abundance. Consistently, comparison of snoRNA / host correlation with previously measured host mRNA halflives (Yang et al 2003) revealed no strict correlation. As expected, ribosomal protein encoding mRNAs were more stable as a group but other host gene mRNAs with similar decay rate did not show the same correlation levels with their embedded snoRNAs. This indicates that the decay rate is not a major determinant of host / snoRNA correlation level. The new comparison is now added in Supplementary Figure S11. We can add that mathematically speaking, unless the decay rate is strongly variable across tissues, we would not expect it to influence the correlation of abundance between host gene and snoRNA. Indeed, decreasing or increasing the abundance of the host gene by a similar proportion in all samples (assuming the decay rate is near constant across tissues) will not change its correlation of abundance with the snoRNA.

We have also addressed in the Background section the fact that AML and cell lines often present methylation systems that are unable to keep up with increased biogenesis, which could explain the discrepancies seen between snoRNA and host gene abundances.

Other comments

Title and other places: The title "Dynamics and modulation of the human snoRNome" and sentences like "to follow the expression of both snoRNAs and HGs in seven healthy human tissues" (p. 5, l. 109) and "dynamics of brain and reproductive tissue snoRNomes ...." (p. 11, l. 244) suggest a time-variable in the study. I have been unable to find justification for this.

Response: To address the reviewer’s comment, we changed the title to “Annotation of snoRNA abundance across human tissues reveals complex snoRNA-host gene relationships”. In addition, we have also removed the notion of time variation from the text.

The use of "snoRNA" as a term throughout leads to the unfortunate situation that RNAs that are not nucleolar are labelled as such. The authors should be explicit about this or, preferably, use better terms, including the established gene names (e.g. SNORD and SNORA).

Response: We agree that the abbreviation snoRNA is unfortunate as increasing evidence indicates roles outside the nucleolus. However, the terms SNORD and SNORA are no better since the NO of snoRNA, SNORD and SNORA all refer to the nucleolus. We have now added a sentence in the introduction indicating that despite their name, not all snoRNAs function in the nucleolus.

The authors should make clear early in the manuscript that they use of the term "expression" in the narrow sense, simply to mean "RNA levels" or, better, use more precise language.

Response: We have now replaced the word ‘expression’ by ‘abundance’ or ‘RNA levels’ throughout the text when appropriate.

p. 3., l. 49 (and other places): "Small nucleolar RNAs (snoRNAs) are a conserved family of mid-size non-coding RNA best characterized as regulators of ribosome biogenesis." There is no evidence that the majority of snoRNAs are "regulatory" or that the installation of a 2'OMe "leads to ribosome assembly" (l. 50). In fact, the literature suggests that most are dispensable. Along the same line, the de-regulation of snoRNA expression in cancer is mostly hypomethylation and can equally well be explained by an inability of the methylation mechanism to keep up with increased ribosome biogenesis. The authors should define their use of the term "regulatory" or, better, avoid using this term loosely.

Response: We have changed the wording in these sentences.

p. 3, l. 54-60: Include acetylation guide RNAs for completion.

Response: We have now added a sentence about snoRNAs guiding acetylation.

p. 4, l. 67: The authors should mention that the example of cis base pairing (ref. 11) may be a special case involved in a regulatory mechanism of snoRNA biogenesis.

Response: We have added the requested information to the sentence.

p. 5, l. 98: The authors should provide evidence for claiming "Indeed, the highly stable and chemically modified structure of snoRNAs impair their reverse transcription, ...". In my opinion, snoRNAs (in particular box C/D) have a limited structural component (e.g. their antisense elements are exposed) and are stabilized by their associated proteins.

Furthermore, the evidence for chemical modifications is very limited. Certainly, the evidence from the Zavolan lab is insufficient to support such a conclusion.

Response: Indeed, there is less evidence supporting chemical modification of snoRNAs resulting in impaired reverse transcription and we have now removed this claim from the sentence. And we agree that box C/D snoRNAs are much less structured than box H/ACA snoRNAs. This concept is stated two sentences after the sentence discussed here.

p. 9, l. 201: "...enrichment of the different genes" should be "gene transcripts" or "transcripts".

Response: The sentence was modified as requested.

p. 13., l. 274-76: The sentence "This suggests that TE snoRNAs have a greater degree of regulatory freedom than the UE group that needs to take in consideration the constraints of the HG expression and function." is unclear to me. What is the meaning of "regulatory freedom"? It seems to me that the authors imply that host genes and snoRNAs (in this case they are most likely not nucleolar and should not be named as such) co-evolve. If so, the authors should provide evidence for this.

Response: This sentence was simply referring to the freedom of the tissue-enriched snoRNA to be regulated independent of their host gene function and was not meant to reflect the evolutionary origin of these snoRNA / host pairs. However, for clarity and to address the reviewer comment we removed the sentence from the text.

p. 14, l. 305-07: "The increased difference between anticorrelated snoRNAs and their HG suggests a negative regulatory loop by which the expression of snoRNAs represses that of the HG." This is highly speculative and should not appear in a results section. Furthermore, what exactly do the authors imply by a "regulatory loop". There is no overall correlation between SNORD levels and modification levels which suggests that SNORD levels not are stringently regulated (or that many SNORDs have additional functions). In several multi-SNORD host genes, the SNORDs have very different abundancies. How is this to be understood in relation to a correlation between

SNORDs and host gene transcript abundancies?

Response: This sentence was meant to raise the possibility and not to confirm the presence of regulatory relationship between the snoRNA and the host genes. Therefore, for clarity we have removed it from the text and refocused our interpretation to the limit of the data presented.

p. 15, l. 334-341: This part was hard to follow. I am quite skeptical about the assignment of functions to many lncRNA and suggest that further arguments are included.

Response: Although it is true that many lncRNAs are non-characterized, the function of some have been studied and these findings are summarized in the database LncTarD. As stated, we used these annotations to label lncRNAs as functional or not. We agree that these are early times for lncRNAs studies but until further characterizations have been carried out, we cannot provide more information or a clearer classification for lncRNAs. We have now modified this section to improve clarity.

p. 16, l. 343: The authors should provide the reader with a short explanation of the background for the data on susceptibility to NMD and should specify if they used the data from the original publication (as referenced) or from the substantial corrigendum that appeared two years later.

Response: We have added a sentence in the Results section explaining how host genes were considered as susceptible to NMD and expanded the explanation of this term in the Methods section. In addition, we also corrected the reference for the substantial corrigendum that was not considered in the original version of the manuscript. We reanalyzed the data with regards to this updated NMD dataset and regenerated the corresponding figures and table (Figures 4D and 5C; Table S5 (previously Table S4)). The new data reinforces the overall conclusions reached in the first version of the manuscript and as such increases the value of the study.

p. 16, l. 360-365: The presentation of the results from ref. 19 on promoter structure is oversimplified. The paper describes two types of initiation sites (YR and YC). Typically each type is used more than once in a promoter. Thus, a promoter is not "single initiation", but "single type initiation" (see e.g. ref. 19, fig. 1b). This also implies that the term "Monolithic initiation" is incorrect! The drawing of the DI promoter in Fig. 6 is incorrect. YR and YC initiation sites are typically closely spaced in the core promoter region (Ref. 19, fig. 1c). In Fig. 6, the YC initiation is drawn much downstream of the YR site and the figure implies that this gives rise to a PTC, which is highly unlikely. A more plausible mechanism for the PTC is alternative splicing which in principle could be linked to different initiation complexes at the two types of TSS, but this is speculative at present.

Response: The language used was meant to reflect the number of different initiation sites and not the number of initiation events as perceived by the reviewer. The use of the term of PTC is meant as the alternative splicing generated signal often linked to NMD and not to imply that PTC results from differences in transcription initiation. However, to avoid confusion, we changed the wording from ‘single initiation’ to ‘simple initiation’ and removed the term monolithic as requested. In addition, we reduced the distance between YC and YR promoters and removed the term PTC from Figure 6.

p. 19, l. 423: I assume that the authors mean "positively correlated" and "single type transcription initiation site".

Response: Indeed, we have changed now modified the text.

Reviewer #2: The article offers highly detailed classification of snoRNAs in a number of human tissues. Overall, the article is well-written and the categories into which the snoRNAs are divided are informative and well-supported. While it is undeniably a valuable resource, the article is purely descriptive. There is no experimental manipulation which would allow for determining the functions of the different snoRNAs. 1.There are certain speculative statements regarding the function of the snoRNAs this article is concerned with in the results section. While some of them are supported by present or previous data, others do not belong in a descriptive article such as this. It would be better to move these statements into the discussion. Examples:

Line 235 - 236: "Together, these data suggest that some snoRNAs may play specialized roles in the biology of brain and reproductive tissues."

Line 243 - 245: "These findings indicate that the distinct nature and dynamics of brain and reproductive tissue snoRNomes may reflect at least in part the clear difference in the proliferative state of the cells forming these two tissue types."

Line 266 - 269: "… indicating different functions associated with the two abundance classes." Etc.

Response: We have removed or modified these and other speculative statements from the text.

2.The figures are crowded due to the limited spacing between the axes and the axes labels. Please adjust the figures so the axes labels are father removed from the axes themselves to improve readability.

Response: We have now moved the axes labels away from the axes and numerical values when possible, in Figures 1C, 2C, 3E-B, 4 and 5A-C.

3.More detail about the individuals the tissues originated from would be helpful. How many were male/female? What age?

Response: We have now included a full description of the tissues’ origin in new supplementary Table S4.

4.What was the minimum RIN score for the samples?

Response: The minimal RIN used was 5.4 and the information is now included in new Table S4.

5.Please report only the p-value in text, without the stars.

Response: We removed all the * from the text, leaving them only in the Figure legends.

6.The percentages in Figure 1 A don't seem to correspond to the total numbers.

Response: The percentages indicated are not the total for all snoRNA but reflect the sum of box C/D or H/ACA snoRNAs separately. We intentionally separated the analysis of C/D and H/ACA snoRNAs to highlight the differences between these two classes and underline the increased proportion of multi-intronic box C/D snoRNA relative to H/ACA snoRNAs. We have clarified this point in the figure legend.

7.Please fix the x axis in Figure 2 C and Figure 4 B.

Response: We now made sure the interval labels are well separated from each other in Figures 2C and 4B.

Reviewer #3: The authors report the application of an RNAseq variant to determine the abundances of snoRNAs across multiple human tissue samples. This leads to conclusions about the gene organization of different groups of snoRNA, which are classified on the basic of their relative accumulation in different tissues. A considerable amount of data are presented, and the analyses appear to have been competently performed with appropriate statistical treatment. Overall, the results are quite descriptive but should be of value to the field. As a class snoRNAs have attracted considerable attention due to correlations with disease states, but their diversity has greatly complicated generalized conclusions. The work reported helps with this significant problem and I would be supportive of publication.

Minor points:

1. For DI vs SI and NMD sensitivity, it would be good to state how these were defined in the main text. These are features used as major classifiers. Where gene lists simply taken from the references cited in Methods?

Response: We added sentences in the Results section explaining how DI vs SI promoters and NMD sensitivity were defined and included more extensive explanation in the Methods section. The DI promoters were identified based on the data generated by Nepal et al. 2020, which classified genes based on the presence of YR and YC promoters using Cap analysis gene expression sequencing (CAGE-Seq). The host genes present in the Supplementary Data 7 of this paper were considered as

containing DI promoters, whereas all other host genes not present in that table were considered as having SI promoters. Host gene susceptibility to NMD was based on the data generated by Lykke-Andersen et al. 2016, which identified genes that are upregulated upon the depletions of NMD factors.

2. In several places there are sentences like: " snoRNAs are generally more expressed than their HG". Gene expression is often used to describe transcription rates. It would be clearer for the reader to distinguish transcription from accumulation, which is actually being measured. The latter is the product of transcription, processing and stability. Some discussion of this point would likely help the reader.

Response: We agree that using the terms ‘abundance’ or ‘RNA levels’ is more appropriate than ‘expression’ and modified the text accordingly.
